# Effect of protective ventilation throughout the intubation period on perioperative oxygenation in patients undergoing MIDCABG: a randomised controlled trial

**DOI:** 10.1080/07853890.2026.2720367

**Published:** 2026-08-26

**Authors:** Min Qian, Zhukai Cong, Ning Yang, Binlong Li, Wenxuan Liu, Changyi Wu, Mao Xu

**Affiliations:** ^a^ Department of Anaesthesiology, Peking University Third Hospital, Beijing, China; ^b^ Beijing Center of Quality Control and Improvement on Clinical Anesthesia, Peking University Third Hospital, Beijing, China

**Keywords:** Protective lung ventilation, minimally invasive direct coronary artery bypass grafting, perioperative oxygenation, lung mechanics, postoperative pulmonary complications

## Abstract

**Introduction:**

Minimally invasive direct coronary artery bypass grafting (MIDCABG) requires prolonged one-lung ventilation (OLV), increasing postoperative pulmonary complications (PPCs) risk. We investigated whether protective lung ventilation (PLV) throughout intubation benefits MIDCABG patients.

**Methods:**

In this single-center randomized study, MIDCABG patients received PLV (low tidal volume of 6–8 mL·kg^−1^, PEEP of 6 cm H_2_O, alveolar recruitment maneuvers) or conventional mechanical ventilation (CMV, tidal volume of 8–10 mL·kg^−1^, without PEEP or maneuvers) from tracheal intubation to extubation. The primary outcome was perioperative oxygenation, assessed by the PaO_2_/FiO_2_ ratio.

**Results:**

Sixty patients (n = 30 per group) were enrolled. Compared with CMV, PLV improved PaO_2_/FiO_2_ ratios (mean difference at OLV_60_: 34.56 mmHg; 95% CI: 11.78–57.33; *p* < 0.01), shortened median durations of postoperative mechanical ventilation (median difference: −4.5 h, 95% CI: −8.5 to −0.5; *p* = 0.013) and hospital stay (median difference: −3.0 days, 95% CI: −5.0 to −1.0; *p* = 0.019). PLV also reduced driving pressure, airway pressure and intrapulmonary shunt during OLV (all *p* < 0.05). Desaturation occurred in 23.3% of CMV patients and 13.3% of PLV patients (*p* = 0.506). Hemodynamic parameters were generally comparable between groups, except for lower MPAP and PVRI in the PLV group during OLV and after ICU admission (*p* < 0.05). The incidence of PPCs did not differ between groups.

**Conclusions:**

In patients undergoing MIDCABG, PLV applied throughout intubation improved perioperative oxygenation and shortened the duration of postoperative mechanical ventilation and hospital stay, but did not reduce PPCs.

**Clinical trial registration:**

ChiCTR1900022005

## Introduction

Minimally invasive direct coronary artery bypass grafting (MIDCABG), performed *via* a left anterior small thoracotomy without cardiopulmonary bypass, has been increasingly adopted worldwide [1,2]. Compared with the conventional mid-sternotomy approach, it is associated with shorter hospital stays, reduced intubation time, and faster postoperative recovery [3,4]. However, this technique requires a prolonged period of one-lung ventilation (OLV) during internal mammary artery harvesting and graft anastomosis.

Postoperative pulmonary complications (PPCs) such as hypoxemia and atelectasis are common after both cardiac surgery [5] and OLV [6]. Because MIDCABG combines the risks of both OLV and cardiac manipulation, pulmonary injury due to mechanical ventilation, alveolar collapse and re-expansion, and surgical trauma is reasonably anticipated [7]. Although PPCs after MIDCABG have not been systematically or comprehensively evaluated, several individual complications including intraoperative hypoxemia [8], prolonged postoperative mechanical ventilation [9], pulmonary edema [10], and impaired lung function at discharge have been reported [11].

MIDCABG also presents a unique challenge: the right ventricle (RV) is subjected to sudden perioperative fluctuations in afterload, preload, and contractility due to heart positioning, protamine administration, and hypoxic pulmonary vasoconstriction (HPV) [12,13]. These changes increase the risk of right ventricular failure and hemodynamic instability, as reported by Mierdl et al. [14].

Protective lung ventilation (PLV) with low VT and moderate PEEP, with or without recruitment maneuvers, has been recommended by experts [15]. Recent studies have demonstrated that PLV can prevent hypoxemia and atelectasis, improve right ventricular performance, and reduce postoperative pulmonary complications following cardiac [5,16] and thoracic surgeries [17,18]. However, intraoperative application of PLV may also induce adverse hemodynamic effects by increasing airway and intrathoracic pressures, particularly in patients with impaired cardiac function [19]. Although different ventilation strategies for minimally invasive cardiac procedures have been explored previously [20,21], no study has directly compared PLV with conventional mechanical ventilation (CMV) as a continuous ventilation strategy throughout the entire intubation period, from the intraoperative period to ICU extubation, in patients undergoing MIDCABG. Moreover, the effects of such a strategy on perioperative oxygenation, clinical outcomes, respiratory mechanics, and hemodynamic stability remain unclear.

Therefore, we designed a randomized controlled trial (RCT) to compare PLV and CMV applied throughout the entire intubation period in patients undergoing MIDCABG. The primary objective was to evaluate the effect of PLV on perioperative oxygenation, assessed by the PaO_2_/FiO_2_ ratio. Secondary objectives included assessment of perioperative oxygenation, clinical outcomes, respiratory mechanics, and hemodynamic stability.

## Patients and methods

### Patients

Inclusion criteria comprised patients aged 18–80 years with American Society of Anesthesiologists (ASA) physical status class I–III scheduled for elective off-pump MIDCABG. All patients underwent routine preoperative hematologic and pulmonary function testing, transthoracic echocardiography, and carotid Doppler ultrasonography. Exclusion criteria included body mass index (BMI) > 40 kg·m^−2^; preoperative shock; emergency surgery; hybrid coronary revascularization; left ventricular ejection fraction < 40%; presence of large pulmonary bullae; chronic obstructive pulmonary disease or active pulmonary infection; neuromuscular disease; perioperative events such as reoperation, intraoperative cardiac arrest, or unplanned conversion to sternotomy; and refusal to participate in the study.

### Randomisation and blinding

After obtaining written informed consent, eligible patients were randomly assigned in a 1:1 ratio to either the PLV group or the CMV group. Randomisation was conducted by an independent individual not involved in the study using an online computer-generated block randomisation system with a fixed block size of 4; allocation was concealed in sequentially numbered, opaque, sealed envelopes. While the anesthesiologist and investigator responsible for perioperative management were aware of group allocation, patients, nursing staff, surgeons, radiologists, and other physicians involved in diagnosing postoperative complications remained blinded to group assignments throughout the study.

### Procedures

Mechanical ventilation was performed using volume-controlled ventilation. The tidal volume (TV) was adjusted according to the patient’s predicted body weight. In the PLV group, TV was set to 8 mL·kg^−1^ during two-lung ventilation (TLV) and reduced to 6 mL·kg^−1^ during OLV, with a positive end-expiratory pressure (PEEP) of 6 cm H_2_O and alveolar recruitment maneuvers (ARM) performed at predefined stages. In the CMV group, TV was set at 10 mL·kg^−1^ during TLV and reduced to 8 mL·kg^−1^ during OLV without PEEP or ARM. Intermittent ARMs were performed at predefined stages as previously described [22]: after intubation, 10 min after skin incision, before LMA anastomosis, and 30 min after ICU admission. ARMs were conducted using pressure-controlled ventilation with stepwise increases in peak inspiratory pressure (PIP) and PEEP (25 cm H_2_O baseline, then 30/10, 35/15, and 40/20 cm H_2_O sequentially). If hemodynamic instability occurred (>20% decrease in MAP or systolic blood pressure < 80 mmHg), 40 µg phenylephrine or 8 µg norepinephrine was administered, with individualized fluid supplementation guided by real-time hemodynamic monitoring. The maneuver was interrupted if hypotension persisted despite adequate fluid and/or vasoactive support. In both groups, FiO_2_ was titrated according to a standardized protocol to maintain SpO_2_ above 92%. During one-lung ventilation, FiO_2_ was generally maintained at ≥ 0.60 to provide an adequate safety margin against hypoxemia. This FiO_2_ titration strategy was identical between the PLV and CMV groups. Desaturation was defined as a single event of SpO_2_ < 92% of any duration during one-lung ventilation.

After surgery, patients were transferred to the ICU and ventilated according to either the CMV or PLV protocol, as determined by randomization. Postoperative management, including sedation, analgesia, fluid therapy, ventilator weaning, extubation, ICU stay duration, and complication management, was performed by ICU physicians according to local protocols. The standard criteria for extubation included hemodynamic stability (no high-dose vasopressors), adequate respiratory effort, absence of significant bleeding, and stable neurologic status. Prophylactic oxygen therapy (3–5 L·min^−1^) *via* nasal cannula was routinely administered after extubation. Pleural drains were routinely removed on postoperative day (POD) 2 or 3. Chest X-rays were obtained on POD 1, POD 3, and POD 5. All patients were followed until hospital discharge.

Perioperative physiological parameters, such as lung mechanics, hemodynamic indices, and blood gas analysis, were recorded simultaneously at the following time points: 30 min after initiation of TLV (TLV_baseline), 30 and 60 min after initiation of OLV (OLV_30_ and OLV_60_), 10 min after resuming TLV (TLV_end), and 4 h after ICU admission (TLV_ICU).

### Primary and secondary endpoints

The primary outcome was perioperative oxygenation, assessed by the PaO_2_/FiO_2_ ratio. PaO_2_/FiO_2_ ratios were measured at predefined perioperative time points to evaluate the effect of the ventilation strategy on oxygenation during MIDCABG.

Secondary clinical outcomes included the incidence of PPCs, duration of postoperative mechanical ventilation, length of ICU stay, and length of hospital stay. PPCs within the first five days were classified and graded according to predefined diagnostic criteria.

Secondary respiratory outcomes included driving pressure (DP), dynamic lung compliance, and pulmonary shunt fraction. Dynamic compliance was calculated as VT/(Pplat − PEEP). Pulmonary shunt fraction was calculated as Qs/Qt.

Hemodynamic parameters were evaluated as safety outcomes because PEEP and recruitment maneuvers may affect venous return, right ventricular afterload, and pulmonary circulation during MIDCABG.

### Statistics

According to a previous meta-analysis [23], it was estimated that a sample size of 30 patients per treatment group would be sufficient to detect a 60 mmHg mean difference in PaO_2_/FiO_2_ between any two groups at the 60-min time point of one-lung ventilation (OLV_60_), with a power of 0.9, a type I error rate of 0.05, and a standard deviation of 30 mmHg. Considering a potential dropout rate of 5%, 33 patients were required for each group. Statistical analyses were performed on a modified intention-to-treat basis, including all patients who were randomized and completed the allocated ventilation protocol.

Normality of data distribution was assessed using the Kolmogorov–Smirnov test. Data are presented as mean (standard deviation, SD) for normally distributed variables or median (interquartile range, IQR) for non-normally distributed variables; categorical data are presented as frequencies and percentages. Demographic, perioperative, and clinical outcome variables were compared between the two groups using the chi-square test or Fisher’s exact test for categorical data, and the independent-samples t-test or Mann–Whitney U test for continuous data. Comparisons of repeated hemodynamic and pulmonary measurements between groups were analyzed using the generalized estimating equation (GEE) method for non-normally distributed data and the general linear model for normally distributed data. Significant temporal changes within groups were analyzed using analysis of variance (ANOVA) or the Friedman test followed by Bonferroni’s post hoc test. Bonferroni correction was applied for multiple comparisons. The durations of postoperative mechanical ventilation, ICU stay, and total hospital stay were compared using Kaplan–Meier curves and log-rank tests. Censoring was performed at 28 days, and time-to-event was defined as the interval from surgery to ICU or hospital discharge. Two-sided P values less than 0.05 were considered statistically significant. All analyses were conducted using SPSS version 25.0 (IBM Corporation, Armonk, NY, USA).

## Results

### Participant characteristics

**Figure 1.**
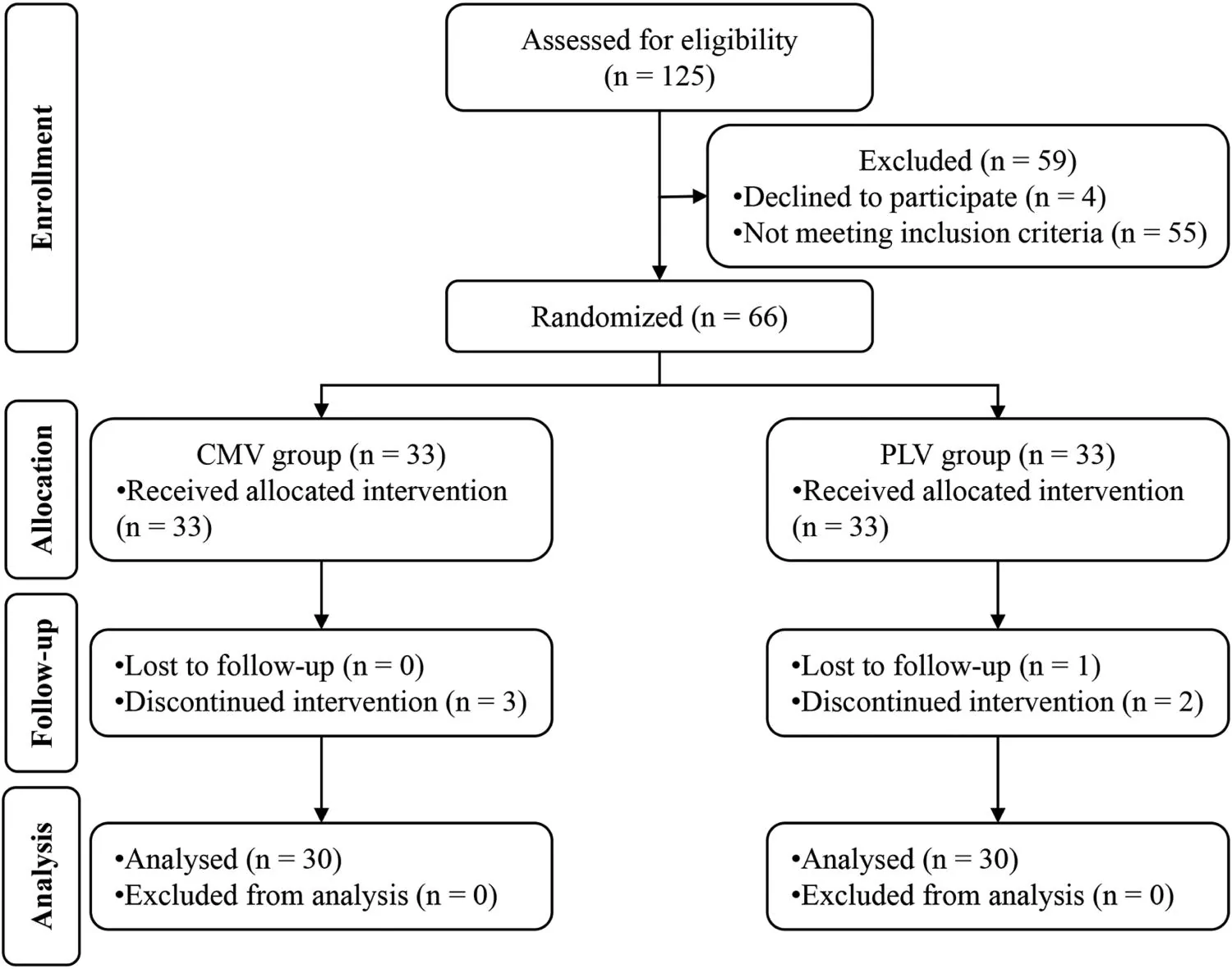
Consolidated Standards of Reporting Trials (CONSORT) flowchart of the study.

From April 2019 to July 2021, 125 patients were assessed for eligibility, and 66 were enrolled in the study. Of these, six patients were excluded from the final analysis: five discontinued the allocated intervention due to a change in the surgical plan, and one was lost to follow-up. Consequently, 30 patients in the CMV group and 30 in the PLV group. The study flow diagram is shown in Figure 1.

Baseline characteristics and preoperative test results, including pulmonary function tests, arterial blood gas analyses, cardiac function, and comorbidities, were comparable between the two groups (Table 1). Intraoperative variables, including sufentanil and rocuronium consumption, total fluid administration, autologous transfusion, use of vasoactive agents, urine output, blood loss, duration of surgery, and duration of mechanical ventilation, did not differ significantly between groups (Table 2).

**Table 1.** Baseline characteristics of subjects stratified by randomization assignment.

Characteristic	CMV (*n* = 30)	PLV (*n* = 30)	Value
Age (yr), mean (SD)	63.2 (9.9)	61.1 (9.7)	0.629
Sex, *n* (%)			0.795
Female	12 (40)	15 (50)	
Male	18 (60)	15 (50)	
Weight (kg), mean (SD)	71.80 (15.88)	67.13 (12.43)	0.21
Height (cm), mean (SD)	166.70 (7.50)	163.37(8.55)	0.114
PBW (kg), mean (SD)	61.0 (8.5)	57.6 (9.6)	0.157
BMI (kg/m^2^), mean (SD)	25.6 (4.0)	25 (3.9)	0.517
ASA physical status, *n* (%)			0.793
I	0	0	
II	17 (56.7%)	13 (43.3%)	
III	18 (60%)	12 (40%)	
Smoking status, *n* (%)			0.844
Never	15 (50%)	13 (43.3%)	
Current	10 (33.3%)	10 (33.3%)	
Former (weaning < 6 month)	5(16.7%)	7 (23.3%)	
Pulmonary function			
FEV1% predicted, mean (SD)	86.2 (13.3)	84.7 (13.7)	0.668
FEV1/FVC% predicted, mean (SD)	92.5 (12.9)	89.9 (11.6)	0.423
PaO_2_ (mmHg), mean (SD)	78.8 (6.1)	77.13 (7.1)	0.323
SaO_2_%, median (IQR)	94 (92, 96)	95 (92, 97)	0.692
Comorbidities, *n* (%)			
Myocardial infarction	10 (33.3%)	7(23.3%)	0.39
Hypertension	15 (50%)	14 (46.7%)	0.432
Stroke	5 (16.7%)	3 (10%)	0.706
Diabetes Mellitus	12 (40%)	10 (33.3%)	0.592
Hyperlipidemia	61 (42.7%)	38 (26.8%)	0.796
Hypertension	18 (90%)	19 (95%)	0.432
Cardiac function			
LVEF (%), median (IQR)	60.5(51.8, 70.0)	62.5(53, 72.3)	0.428
EuroSCORE II %, median (IQR)	0.77 (0.53, 1.18)	0.88 (0.61, 1.12)	0.911
NYHA function class, *n* (%)			0.647
I	16 (53.3%)	20 (66.7%)	
II	10 (33.3%)	7 (23.3%)	
III	4 (13.3%)	3 (10%)	
IV	0	0	

Data are presented as mean (standard deviation, SD), median (interquartile range, IQR), or *n* (%). BMI, body mass index (kg·m^2^); ASA, American Society of Anesthesiologists; LVEF, left ventricular ejection fraction; FEV_1_, forced expiratory volume in one second; FEV_1_ (%), percentage of predicted forced expiratory volume in one second; PaO_2_, partial pressure of oxygen in arterial blood; SaO_2_, arterial oxygen saturation; NYHA, New York heart association classification.

**Table 2.** Comparison of intraoperative characteristics between the two groups.

Variables	CMV (*n* = 30)	PLV (*n* = 30)	*p* Value
Duration of anesthesia (min), mean (SD)	406.3 (111.7)	381.4 (93.8)	0.35365
Duration of surgery (min), mean (SD)	313.1 (107.3)	289.0(87.8)	0.34396
Duration of one-lung ventilation (min), mean (SD)	194.6 (59.8)	174.3 (58.5)	0.18964
Total amount of rocuronium (mg), median (IQR)	171.5 (152.5, 207.5)	170 (140, 200)	0.259
Total amount of sufentanil (µg), median (IQR)	177.5 (157.5, 202.5)	165 (147.5, 196.2)	0.239
Total amount of crystalloid (mL), median (IQR)	2200 (2175, 2700)	2200 (1850, 2700)	1
Total amount of colloid (mL), median (IQR)	250(0, 500)	250(0, 500)	1
Total amount of autologous transfusion, median (IQR)	205(120, 350)	177(122, 256)	0.446
Urine output (mL), median (IQR)	700 (337, 800)	500 (375, 712)	0.289
Bleeding (mL), median (IQR)	420 (240, 800)	380 (240, 570)	0.407
Fluid balance (mL), median (IQR)	1688 (1495, 1878)	1615(1276, 2180)	0.728
Numbles of grafts, median (IQR)	3 (2, 3)	2.5 (2, 3)	0.641
Norepinephrine (µg), median (IQR)	110 (15, 420)	90 (8, 400)	0.982
Phenylephrine (µg), median (IQR)	360 (100, 480)	400 (160, 600)	0.451
Nitroglycerin (mg), median (IQR)	215 (67.5, 400)	180 (37.5, 340)	0.328
Dopamine (mg), median (IQR)	3 (0, 10)	4 (0.75, 8)	0.663

Data are presented as mean (standard deviation, SD), median (interquartile range, IQR), or *n* (%).

### Primary outcomes

**Figure 2.**
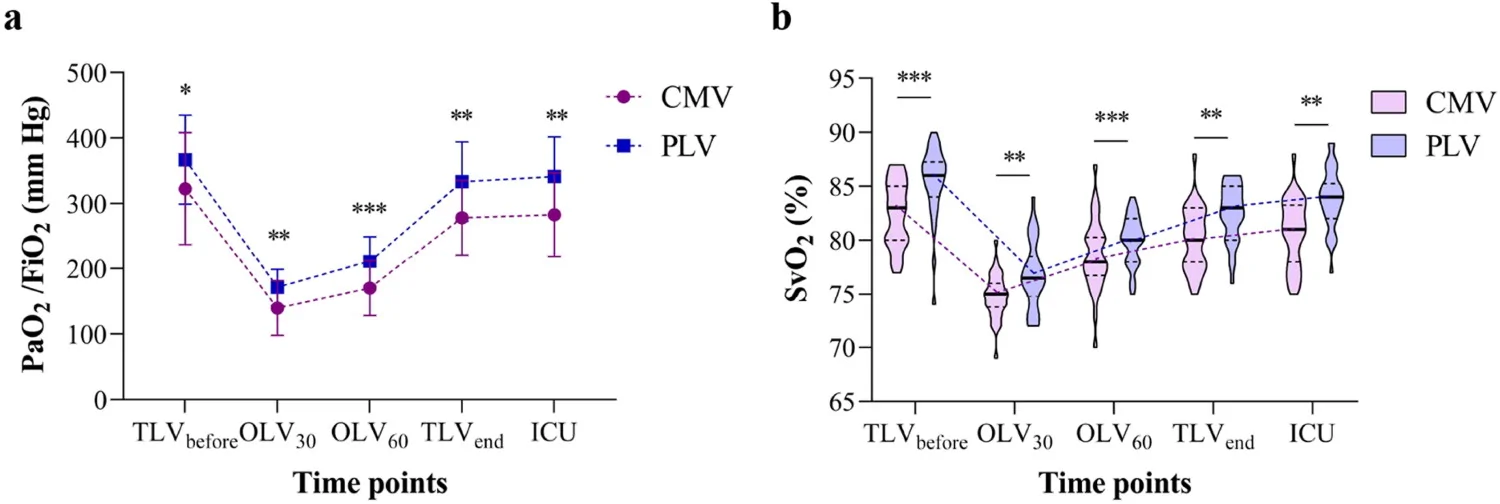
Intraoperative and postoperative blood gas analyses. (a) PaO_2_/FiO_2_; (b) SvO_2_. PaO_2_, arterial oxygen pressure; SvO_2_, mixed venous oxygen saturation; TLV_before_, TLV before surgery; OLV_30_ and OLV_60_, 30 and 60 min after OLV; TLV_end_, 10 min after TLV re-initiation. Variables are presented as mean (standard deviation, SD), median (interquartile range, IQR), or *n* (%), according to data distribution.

PaO_2_/FiO_2_ ratios decreased during OLV in both groups compared with TLV baseline (Figure 2a, *p* < 0.001). However, PaO_2_/FiO_2_ ratios remained consistently higher in the PLV group than in the CMV group throughout the observation period with a particularly pronounced difference at OLV_60_ (mean difference: 34.56 mmHg; 95% CI: 11.78 to 57.33; *p* < 0.01). After the resumption of two-lung ventilation, PaO_2_/FiO_2_ returned to baseline at TLV_end in the PLV group and at TLV_ICU in the CMV group. SvO_2_ decreased during OLV in both groups and recovered after ICU admission, with higher values observed in the PLV group across the observation period (Figure 2a, *p* < 0.01). Desaturation, defined as SpO_2_ < 92% during OLV, occurred in 23.3% of patients in the CMV group and 13.3% in the PLV group, with no significant between-group difference (Table 3, *p* = 0.506).

**Table 3.** Intraoperative and postoperative outcomes.

Intraoperative events and postoperative outcomes	CMV (*n* = 30)	PLV (*n* = 30)	*p* Value
Intraoperative hypoxemia, *n* (%)	7 (23.3%)	4 (13.3%)	0.506
Mechanical ventilation duration (hours), median (IQR)	14 (10, 18)	9 (6, 14)	0.013
Intensive care unit stay (hours), median (IQR)	24 (16.8, 45)	22.5 (12, 27)	0.063
Hospital stays (days), median (IQR)	19 (14.8,22.2)	16 (13, 18.5)	0.019
Post-operative hospital stays (days), median (IQR)	8 (7, 1)	7 (6, 8.2)	0.013
Supplemental O_2_ > 48h, *n* (%)	11 (36.7%)	4 (13.3%)	0.072
Ventilator support > 48h, *n* (%)	2 (6.9%)	0	0.273
Postoperative pulmonary complications, *n* (%)	27 (90.0%)	28 (93.3%)	1
Early respiratory failure, *n* (%)	5 (16.7%)	5 (3.3%)	0.197
Left pleural effusion, *n* (%)	25 (83.3%)	24 (80%)	0.731
Right pleural effusion, *n* (%)	13 (44.8%)	17 (56.7%)	0.439
Left pneumothorax, *n* (%)	9 (30%)	7 (23.3%)	0.561
Right pneumothorax, *n* (%)	0	1 (3.3%)	/
Pneumonia, *n* (%)	2 (6.7%)	1 (3.3%)	1
Reintubation, *n* (%)	1 (3.3%)	0	/
Atelectasis, *n* (%)	9 (30%)	7 (23.3%)	0.771
Pulmonary complication severity score, median (IQR)	2 (2, 2)	1.5 (1, 2)	0.186
VAS score	3 (2, 4)	3 (3, 4)	0.604
Other outcomes			
Stroke	0	0	/
Hospital wound infection,	0	0	/
Myocardial infarction, *n* (%)	1 (3.3%)	1 (3.3%)	/
Atrial arrhythmia, *n* (%)	0	1 (3.3%)	/

VAS, visual analogue scale. Variables are presented as median (interquartile range, IQR) or *n* (%).

### Secondary clinical outcomes

**Figure 3.**
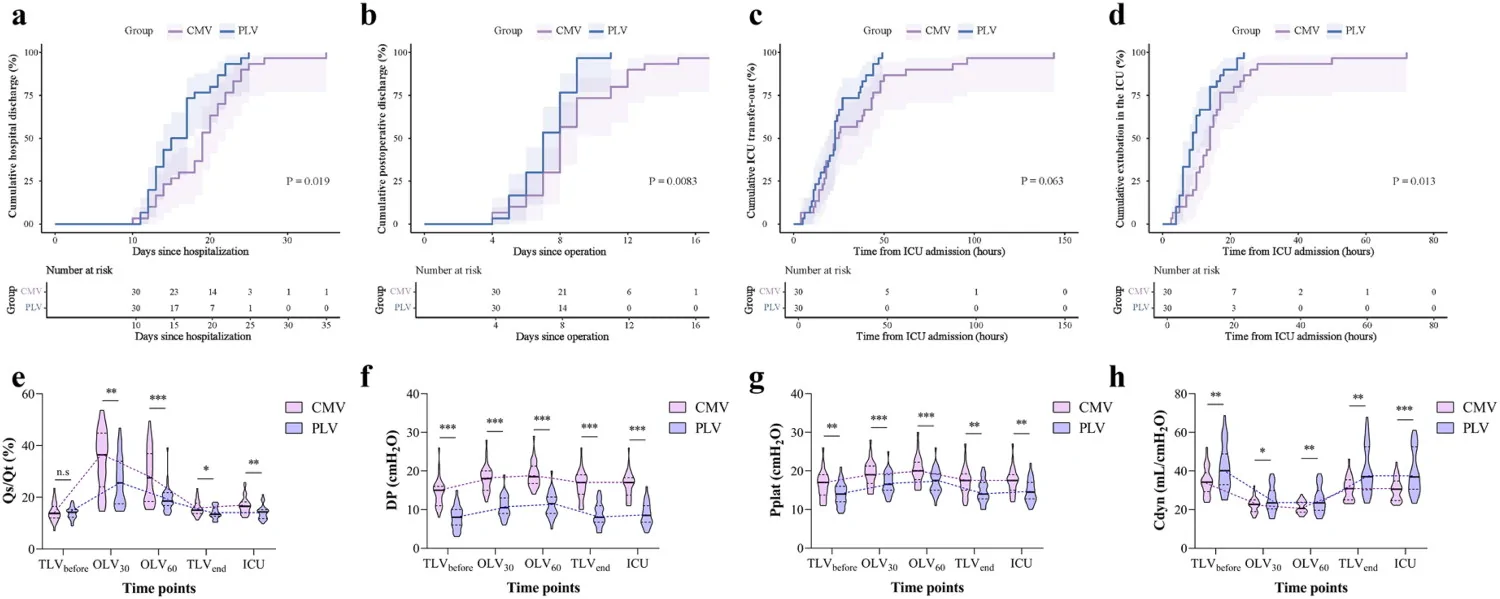
Kaplan–Meier curves showing (a) hospital stay (days), (b) postoperative hospital stay (days), (c) mechanical ventilation duration (hours), and (d) intensive care unit stay (hours) in the CMV and PLV groups. Intraoperative and postoperative pulmonary mechanics (e) Qs/Qt; (f) DP; (g) PAP; (h) Cdyn. Qs/Qt, intrapulmonary shunt fraction; PAP, peak airway pressure; Pplat, plateau pressure; Cdyn, pulmonary dynamic compliance. Variables are presented as mean (standard deviation, SD), median (interquartile range, IQR), or *n* (%), according to data distribution.

Secondary clinical outcomes are summarized in Figure 3 and Table 3. No mortality occurred in either group. Compared with the CMV group, the PLV group had a shorter median hospital stay (median difference: −3.0 days, 95% CI: −5.0 to −1.0; *p* = 0.019), shorter time from surgery to discharge (median ­difference: −1.0 day; 95% CI: −2.0 to 0.0; *p* = 0.0083), and shorter duration of postoperative mechanical ventilation (median difference: −4.5 h, 95% CI: −8.5 to −0.5; *p* = 0.013). ICU length of stay did not differ significantly between groups. The incidence and severity of PPCs within the first 5 postoperative days were comparable between groups, as were the individual PPC components, including atelectasis, pleural effusion, pneumothorax, and pneumonia (Table 3, *p* = 1.00). No patient in the PLV group required reintubation, whereas one patient in the CMV group did. Postoperative myocardial ischemia occurred in one patient in each group.

### Respiratory mechanics, pulmonary shunt, and hemodynamic outcomes

Respiratory mechanics and pulmonary shunt data are shown in Figure 3. Qs/Qt increased during OLV in both groups, but the increase was smaller in the PLV group than in the CMV group (*p* < 0.05). Qs/Qt returned to baseline by the end of surgery in the PLV group, whereas it remained elevated after ICU admission in the CMV group. Both DP and PAP increased after the initiation of OLV, but were consistently lower in the PLV group throughout the observation period (*p* < 0.01). Dynamic compliance decreased after the transition from TLV to OLV in both groups and recovered after lung re-expansion, with no significant between-group difference.

Hemodynamic variables are shown in Supplementary Figure 1. Overall, PLV did not cause clinically relevant hemodynamic compromise. HR, CVP, CI, PCWP, and SVRI were comparable between groups throughout the study period. MPAP and PVRI increased during OLV in both groups, but were lower in the PLV group at selected time points during OLV and after ICU admission. CI was maintained throughout the observation period in both groups.

## Discussion

The principal finding of the study is that, in MIDCABG, the application of a PLV strategy, compared with CMV without RM and PEEP, significantly improved oxygenation and lung mechanical properties without inducing significant hemodynamic effects. Patients in the PLV group also experienced shorter hospital stays and reduced ICU ventilation durations, although no significant differences were observed in ICU stay length or PPCs incidence.

The use of MIDCABG has expanded with surgical advances, providing a less invasive off‑pump alternative to open‑chest procedures. However, OLV is typically required for a substantial portion of this surgery, resulting in decreased lung volume, reduced lung compliance, and increased intrapulmonary shunting [24]. Desaturation (SpO_2_ < 92%) occurred in 23.3% of CMV and 13.3% of PLV patients (*p* = 0.506), which was higher than the 4–10% hypoxia rate (SpO_2_ < 90%) reported in lateral-decubitus thoracic surgery [25]. This discrepancy may be due to the semi‑lateral or supine positions used in MIDCABG, which limit gravity‑induced redistribution of pulmonary perfusion to the ventilated lung, thus increasing shunt and hypoxemia [26]. In a study by Liu et al. which evaluated oxygenation during OLV in the supine position for robot-assisted CABG, 12.6% of patients experienced hypoxemic events (PaO_2_ < 9.31 kPa) during passive pneumothorax [8]. OLV tolerance appeared higher in our cohort, possibly because the aforementioned study included more patients with chronic obstructive pulmonary disease.

Several clinical studies in thoracic and cardiac surgery have reported improved oxygenation and ventilation following ARM combined with moderate PEEP (5–10 cm·H_2_O) [13,15,23]. Consistent with these findings, the implementation of PLV in our study enhanced arterial oxygenation and dynamic compliance, and reduced Qs/Qt during OLV and the early postoperative period compared with CMV. These effects may be attributed to the attenuation of atelectasis due to the combined effects of reexpansion maneuvers and PEEP, which help maintain alveolar patency, as demonstrated in previous studies [27,28]. The higher tidal volume strategy in the CMV group resulted in significantly higher driving and airway pressures. This elevated transpulmonary pressure may have contributed to alveolar overdistension and capillary compression, increasing PVR. Furthermore, the higher intrapulmonary shunt observed in the CMV group suggests more severe alveolar collapse, which can trigger a more pronounced HPV in the non-ventilated lung. Both mechanisms could explain the higher MPAP and PVRI observed in the CMV group. Conversely, while PEEP in the PLV group can theoretically increase PVR, the beneficial effects of alveolar recruitment in mitigating atelectasis and HPV appear to have predominated, leading to a net reduction in right ventricular afterload. Previous studies have identified increased DP as a predictor of pulmonary complications following cardiac and thoracic surgery [29,30], while inconclusive findings in cardiothoracic surgery suggest that DP alone may be insufficient as a ventilation target [31]. Elevated PAP at the end of surgery has been recognized as a risk factor for prolonged mechanical ventilation after robot-assisted CABG [9]. Other ventilatory parameters should not be overlooked as they may be implicated in lung stress, suggesting increased damage from biotrauma with large VT [28].

Transient hemodynamic instability during ARM is common in cardiothoracic surgery but rapidly reversible upon termination [3,12,27], and may be exacerbated by frequent cardiac displacement during MIDCABG. To minimize this, we optimized fluid administration and used a stepwise ARM protocol instead of sustained inflation. Consequently, HR, MAP, and CI did not differ significantly between groups, and requirements for fluids and vasoactive drugs were comparable. Right ventricular impedance is another concern. During OLV, lung collapse increases pulmonary vascular resistance (PVR) and right ventricular workload due to activation of HPV [32]. ARM and high PEEP may elevate transpulmonary pressure and compress pulmonary capillaries, thereby decreasing right ventricular preload and increasing afterload [12]. However, stepwise ARM reversed the adverse hemodynamic effects in ARDS patients by resolving atelectasis [19]. Longo et al. demonstrated that atelectasis after cardiac surgery impaired right ventricular function, which was restored by lung recruitment using 10 cmH_2_O PEEP, as confirmed by transesophageal echocardiography [33]. Using Swan-Ganz catheterization, we found greater PVRI increase in CMV than PLV after OLV. Together with the improvements in pulmonary compliance and oxygenation, these findings suggest that atelectasis following OLV can be alleviated by PLV in our study.

This improvement in gas exchange and pulmonary mechanics during mechanical ventilation enabled earlier extubation and shorter hospital stays for patients in the PLV group. The observed severity distribution of PPCs was similar to that reported by Costa et al. [16]. However, we were unable to demonstrate that protective ventilation strategies were associated with a reduced incidence of PPCs in patients with normal lungs after MIDCABG. Previous studies investigating the effects of PLV on PPCs during the postoperative course of CABG mainly focused on patients undergoing on-pump CABG and yielded inconclusive results [16,34]. One study reported that PLV after off-pump CABG shortened intubation and hospitalization durations, with fewer respiratory complications such as atelectasis and pleural effusion [35]. However, the PEEP level in that study was set at 10 cmH_2_O. In contrast, we selected a PEEP of 6 cmH_2_O for defining protective OLV, based on published expert recommendations, which may have been insufficient to maintain an open lung state and prevent atelectasis [36]. Moreover, the incidences of atelectasis and pleural effusion were markedly higher in our cohort, possibly due to increased surgical trauma involving direct lung manipulation. Pleural effusion after cardiac surgery has been associated with impaired oxygenation and reduced pulmonary function [37].

Several limitations of the present study should be acknowledged. First, individualized PEEP titration was omitted due to lack of validated methods and concerns about hemodynamic intolerance and barotrauma [36]. Second, intrinsic PEEP unmeasurable by the ventilator, may have biased driving pressure estimates [31]. Third, the incidence of PPCs was unexpectedly high in both groups (90.0% vs 93.3%). This is largely attributable to the high rates of radiological atelectasis and pleural effusion, which are common sequelae of thoracic surgery and may not always represent clinically significant pathology. The incidence of more severe complications like pneumonia and pneumothorax was low and comparable between groups. This suggests that while PLV may improve physiology, it may not be sufficient to prevent these less severe, surgically-related radiological findings. Fourth, this was a single-center study, which may limit the generalizability of our findings. The study protocol primarily aimed to evaluate respiratory effects, and the predefined minimum clinically meaningful sample size may have limited the interpretation of clinical outcomes. Future multicenter trials investigating specific lung-protective ventilation strategies to improve clinical outcomes after MIDCABG are warranted.

## Conclusions

In patients undergoing MIDCABG, a continuous PLV strategy improved perioperative oxygenation and lung mechanics compared to CMV, without significant hemodynamic compromise. This strategy was also associated with an exploratory, albeit shorter, duration of postoperative mechanical ventilation and hospital stay. However, it did not significantly reduce the incidence of PPCs.

## Supplementary Material

Supplemental Material

Supp.jpg

## Data Availability

The data which support this study can be obtained from the corresponding authors on reasonable request.
