# Supplementary material for: Effect of protective ventilation throughout the intubation period on perioperative oxygenation in patients undergoing MIDCABG: a randomised controlled trial
Source: Ann Med. 2026 Aug 26;58(1):2720367. doi: 10.1080/07853890.2026.2720367 (PMC13523107; doi:10.1080/07853890.2026.2720367)
Supplement: Supplementary file 1 [file IANN_A_2720367_SM7658.docx]

**Supplementary Figure 1.** Intraoperative and postoperative hemodynamic parameters (a) HR; (b) MAP; (c) PCWP; (d) CVP; (e) MPAP; (f) CI; (g) SVRI; (h) PVRI. MAP, mean arterial pressure (mmHg); MPAP, mean pulmonary artery pressure (mmHg); CVP, central venous pressure (mmHg); PCWP, pulmonary capillary wedge pressure (mmHg); HR, heart rate (beats per minute); SVRI, systemic vascular resistance (dyn·s·cm⁻^5^·m^2^); PVRI, pulmonary vascular resistance (dyn·s·cm⁻^5^·m^2^).
